# Inferring functional brain connectivity from field-potential oscillations in health and disease

**DOI:** 10.1186/1471-2202-12-S1-P108

**Published:** 2011-07-18

**Authors:** G Karl Steinke, Roberto F Galán

**Affiliations:** 1 Department of Biomedical Engineering, Case Western Reserve University, Cleveland, OH 44106, USA; 2 Currently working at Boston Scientific Neuromodulation, Valencia, CA 91355, USA; 3 Department of Neurosciences, Case Western Reserve University, Cleveland, OH 44106, USA

## 

**Figure 1:**
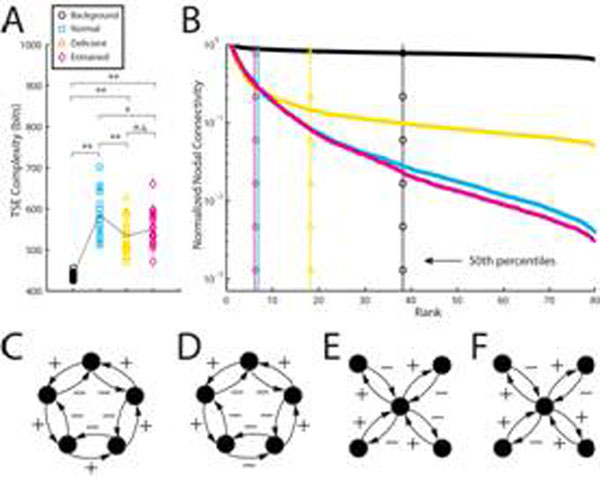
Structural properties of virtual brains from four groups with different power spectra (Background: 1/f spectrum; Normal; Deficient: missing high frequency content – putative schizophrenic brain; Entrained: frequency bands are rational multiples of each other – putative schizophrenic brain). **A)** Neural complexity is significantly reduced in groups modeling pathological cases. **B)** Ranking of nodes according to their connectivity reveals a hierarchical brain organization that is less pronounced in the Deficient group. **C)** Example of an under-represented motif in both pathological cases, **D)** of an over-represented motif in the Entrained group, **E)** of an under-represented motif in the Normal group, and **F)** of an over-represented motif in the Deficient group.

Field-potential recordings (e.g. EEG, MEG) of ongoing neural activity exhibit oscillations of specific frequencies over a pink-noise 1/f background [1]. The oscillations appear in the power spectrum as a collection of frequency bands evenly spaced on a logarithmic scale, thereby preventing mutual entrainment and cross-talk. Applying mathematical techniques for inverse problems [2], we reverse-engineered network architectures with 80 nodes that generate these characteristic dynamics of normal brain function. We show that all reconstructed networks, or “virtual brains”, display similar topological features (e.g. structural motifs) and dynamics (e.g. spindle and sharp waves). We also reverse-engineered putative diseased brains (epileptic and schizophrenic), in which oscillatory activity is altered in different ways [3]. The reconstructed networks show consistent alterations of functional connectivity and dynamics. These alterations lead to a decrease in neural complexity (Fig. 1A), as defined in [4], changes in the hierarchical structure of the brain connectivity (Fig. 1B) and in the probability of finding certain structural motifs (Figs. 1C1D1E1F). The predictions from our model may be easily tested in actual brains.
